# Multilayer perceptron neural network approach for power quality improvement in a grid integrated PV and electric vehicle systems

**DOI:** 10.1371/journal.pone.0350947

**Published:** 2026-06-11

**Authors:** Soumya Ranjan Das, Jayalakshmi Narayana Sabhahit, K. Abhimanyu Kumar Patro, Devi Prasad Acharya, Asit Mohanty, Erdem Cuce

**Affiliations:** 1 Manipal Institute of Technology, Manipal Academy of Higher Education, Manipal, Karnataka, India; 2 Department of EE, Parala Maharaja Engineering College, Berhampur, Odisha, India; 3 Centre for Promotion of Research, Graphic Era (Deemed to be University), Dehradun, India; 4 Faculty of Engineering and Architecture, Recep Tayyip Erdogan University, Rize, Türkiye; Dr Shakuntala Misra National Rehabilitation University, INDIA

## Abstract

Recently, there has been an increase in the grid integration of electric vehicles (EVs) and solar photovoltaic (PV) systems, primarily driven by two goals: lowering energy costs and decreasing emissions. Numerous research studies have concentrated on the separate effects of integrating PVs and EVs into the grid. Nevertheless, it is important to recognize that as the adoption of PVs and EVs continues to grow, the supply grid will face the cumulative effects of PV and EV integration on power quality (PQ) challenges. To provide a comprehensive understanding, this study examines the joint impact of PVs and EVs on PQ aspects in detail. This study has indicated that EVs and PVs alone can adversely impact grid reliability and PQ because of the variable character of PV source and the unpredictability of EV demand. But multiple research efforts have shown that coordination between PVs and EVs can help to alleviate certain problems that arise from their individual integration. This study demonstrates PQ enhancement in a grid system integrated with PV and EV using a multilayer perceptron neural network (MLPNN) approach. In the system with PV integration, the GWO-ANFIS, MPPT technique is employed for optimizing power extraction. Under balanced non-linear loading conditions, simulation results show that the THD is initially 25.97% without compensation, then decreases to 12.57% with a shunt passive filter (SPF), 3.37% with the application of recursive least squares (RLS), and 1.37% with MLPNN. With much lower THD and quicker convergence, the suggested MLPNN-based controller exhibits improved harmonic mitigation. A comparison between the proposed and existing methods are drawn using the MATLAB/Simulink platform.

## Introduction

The global energy landscape is undergoing a significant transformation driven by the increasing demand for clean, sustainable, and efficient energy solutions. Traditionally, electrical power systems were designed around centralized generation units such as thermal, hydro, and nuclear power plants where, power flows unidirectionally from generation to transmission and finally to consumers. These conventional systems were relatively predictable, allowing stable operation with well-maintained voltage, frequency, and waveform characteristics. Nevertheless, in the recent past, a paradigm shift has occurred towards the use of renewable and environment-friendly sources of energy. Some of the renewable energies that are highly scalable, operate at lower costs, and cause minimum environmental damage include PV systems. In addition, the transport industry has experienced high electrification rates, particularly using EVs that minimize the use of fossil fuel energy sources and reduce greenhouse gases.

Although the use of PV systems together with EVs provides many environmental and economic advantages and helps to meet sustainable energy needs while reducing carbon footprints, it also creates some technical problems for PQ [1].

Unlike conventional systems, both PV generation and EV charging rely heavily on power electronic converters [2], which inherently exhibit non-linear and dynamic characteristics. Also, PV systems are inherently variable sources, whose generation is dependent on sunshine and weather, while EV charging is a random process based on the requirements of the users. As a result, the maintenance of PQ parameters such as voltage, frequency, harmonics, and waveform, among others, has become difficult.

Several recent literature reviews have been published about the effect of the integration of PV-EVs on PQ problems. The large-scale deployment of photovoltaic systems and electric vehicles has posed serious questions on the effect of these systems on PQ problems like voltage fluctuation, harmonic distortion, voltage boost, frequency deviation, and load variation. Additionally, bidirectional power flow is introduced by quick EV charging and vehicle-to-grid (V2G) operations, which further complicates system management and control. The necessity of coordinated communication between transportation networks and power distribution systems has been highlighted by recent developments in EV integration. In order to maximize system performance, a number of studies have investigated integrated EV-transportation-grid frameworks that take charging demand and traffic flow models into account [3]. Moreover, optimization algorithms as charging schemes, involving multi-objective optimizations as well, have been extensively used to optimize grid stability, cost of charging, and the use of renewable resources [4]. With respect to the integration of RE resources, especially photovoltaic systems, in combination with V2G technology, EVs have been turned into flexible storage systems for peak load control and increased grid flexibility [5]. In addition, recent advances in data-driven and real-time scheduling methods have made the EV load forecasting and control more accurate [3,4]. However, there is little concern on PQ problems, like harmonics and voltage distortion in PV-EV systems.

While there have been extensive studies done regarding EV charging optimization, grid transportation integration, and renewable energy coordination [3,5], many current studies do not address power quality problems such as harmonic distortion, unbalanced voltages, and distorted waveforms in PV-EV distribution networks. According to a thorough study conducted in the field, EV charging stations cause harmonic distortions and variations in voltages, thus requiring an effective solution to the problem [6]. In the same way, a recent study conducted on PV-based smart grids has found that ensuring stability is a vital factor [7] to consider.

To overcome these issues, several researchers have suggested intelligent and hybrid control systems. For example, in PV-powered EV charging systems, hybrid control methods that combine fuzzy logic and sliding mode control have shown notable gains in lowering total harmonic distortion (THD) and preserving voltage stability [8]. Additionally, it has been demonstrated that sophisticated optimization-based controllers combined with disturbance rejection methods improve system efficiency and reduce power loss under a variety of load scenarios [9]. Moreover, research related to EV-based microgrids shows that optimal load scheduling and renewable energy integration techniques can effectively decrease harmonic distortion and enhance voltage quality particularly at partial load operation [10].

Despite these advancements, existing works often treat PV systems and EV loads independently or rely on conventional control methods, limiting their effectiveness under real-time dynamic scenarios. Solar irradiance intermittency in PV power systems with integrated EV charging functionality is shown in [11]. The design of an EV charging station with solar PV integration and its PQ effect is shown in [12]. The active and reactive power, efficiency, reliability, and voltage support needs can all be influenced by the grid-connected PV-EV system [13]. The grid connects the PV and EV through various power converters. Utilizing a bi-directional EV charger connected to the DC connection of the solar inverter can reduce the acceleration of PV power supply and provide rapid battery charging from the PV system [14,15].

The integration of PV and EV systems requires advanced control strategy to maintain voltage, frequency, and PQ. In this work, a voltage source inverter (VSI) based shunt hybrid active power filter (SHAPF). Therefore, different control strategies are required for maximum power point tracking (MPPT) [16] in solar PV system and current control in VSI. Several research analyses have been conducted to observe MPPT to maximize output power from PV under irradiation conditions. Some of them are discussed like conventional MPPTs, such as the Perturb and Observe method [17,18], the incremental conductance technique [19], the constant voltage algorithm [20,21], the hill climbing algorithm [22,23], as well as conventional metaheuristic controllers like standard fuzzy logic controllers [24,25] and neural networks [26,27]. Some new meta-heuristic optimization techniques like global maximum power point and issues related to partial shading are addressed in [28]. Some other optimization-based methods, like particle swarm optimization [29] and artificial bee colony [30], has been utilized in the past to enhance the efficiency of PV panels. Nevertheless, these approaches do not consistently outperform each other when used independently. Hybrid metaheuristic algorithms can address this challenge and produce constructive outcomes for MPPT purpose. Similarly, Fuzzy Logic Controller (FLC) with grasshopper-based optimization for solar PV systems to handle specific variations in temperature and irradiance [31], along with a combination of differential evaluation and particle swarm optimization [32], and a whale optimization method for MPPT controllers [33], as well as various other genetic algorithms documented in the literature [34–37]. Conventional MPPT techniques suffer from oscillations and reduced efficiency under rapidly changing environmental conditions. Although metaheuristic and hybrid optimization techniques improve tracking performance, they often introduce increased computational complexity.

In this paper, for performing the MPPT, the GWO-ANFIS is used to track maximum power. The solar PV and EV are integrated with the grid using grid connected VSI. Therefore, various control methods are discussed for controlling the voltage and current. The methods for harmonics extraction can be categorized into three groups: frequency, time domain, and artificial intelligence approaches. The former is typically linked to the calculation of Fourier coefficients and the time lag in sampling leads to a major challenge in every technique under this section. The time domain generally exhibits superior performance concerning convergence speed when compared to the frequency domain. However, there are concerns about potential flickers and noise that may arise from the transformation of coordinates from the input signals.

Furthermore, the application of intelligent control techniques, particularly neural network-based approaches, for real-time PQ enhancement remains relatively unexplored. Artificial neural network (ANN) represents one of the artificial intelligence methodologies employed to reduce harmonic components and can effectively estimate or extract time-varying fundamental parts regarding phase angle and magnitude.

Several adaptive controllers have been proposed in [38,39] for controlling harmonic currents through SHAPF. The most common adaptive controllers employed in this context are the least mean square (LMS) algorithm [40] and the recursive least square (RLS) algorithm [41,42]. These two adaptive controllers are popular because of their superior efficiency, simple implementation, reliability, and negligible computational complexity. Nevertheless, although the design of these controllers is easy and the implementation is efficient, the speed of convergence is rather poor. The RLS converges much faster than the LMS, and the performance of the latter filter is much better than that of the former.

In addition to LMS and RLS methods, there are also many other methods like ANFIS [43], hybrid GWO-ANFIS [44], which are widely applied in efficient harmonics mitigation and need much fine-tuning and computational load. To overcome these limitations, MLPNN [45,46] based control method has been employed in the present study, in which each artificial neuron uses a non-linear differentiable activation function, where synaptic weight signifies highly connected neurons. The control method guarantees efficient harmonics cancellation, ensuring that THD of source current lies within IEEE-519 standards and achieves almost unity power factor with non-linear loads. A comparative approach is shown in Table 1.

**Table 1:** Comparative analysis of different control methods.

Method	Category	Convergence Speed	Computational Burden	Key Observation
LMS	Adaptive Filter	Slow	Low	Simple but slow convergence
RLS	Adaptive Filter	Moderate	High	Faster but computationally intensive
ANFIS	Intelligent Control	Moderate	Moderate	Improved accuracy, requires training
GWO-ANFIS	Hybrid Optimization	Fast	High	Handles nonlinearities effectively
Proposed MLPNN	Neural Network	Fast	Moderate	Best trade-off, real-time feasible

Despite significant advancements in PV-integrated systems and EV charging technologies, several limitations remain in existing literature. Existing works lack a unified control strategy capable of simultaneously handling PV intermittency, EV load variability, and harmonic mitigation under dynamic operating conditions. This highlights the need for a more robust and adaptive control framework. Therefore, there exists a research gap in developing an integrated, computationally efficient, and adaptive control strategy that can effectively address PQ issues arising from the combined operation of PV and EV systems while ensuring fast convergence and reduced harmonic distortion.

Unlike traditional methods where independent adaptation filters or neural networks are used, the presented technique makes use of a novel intelligent controller design based on ANFIS and MLPNN to achieve MPPT and harmonics reduction in the grid integrated PV-EV system. The presented methodology offers enhanced convergence properties and lower harmonic distortion in addition to being more adaptable compared to traditional LMS and RLS techniques.

The main contributions of this work are summarized as follows:

1. A novel hybrid PV–EV integrated PQ improvement framework is proposed, addressing the combined impact of solar PV and EV charging loads on distribution systems.
2. A dual intelligent control strategy is developed by integrating: (a) GWO-ANFIS-based MPPT for optimal power extraction and (b) MLPNN-based adaptive filtering for harmonic mitigation and reference current generation.
3. The proposed MLPNN controller overcomes the limitations of conventional adaptive filters by providing faster convergence, reduced steady-state error and lower computational complexity compared to RLS.

The proposed approach advances the state-of-the-art by offering a robust, adaptive, and computationally efficient solution for PQ management in modern hybrid RE systems. The proposed approach is employed using the MATLAB/Simulink tool, and the outcomes are compared with conventional algorithms. The proposed system is validated through real-time implementation using dSPACE 1103, demonstrating its practical feasibility for real-world applications. The proposed research supports the sustainable development goals (SDG); SDG 7 and SDG 13 providing clean energy by improving the integration and efficiency of RE systems and encouraging low-carbon energy solutions.

## System design of the proposed PV-EV integrated system

**Fig 1:**
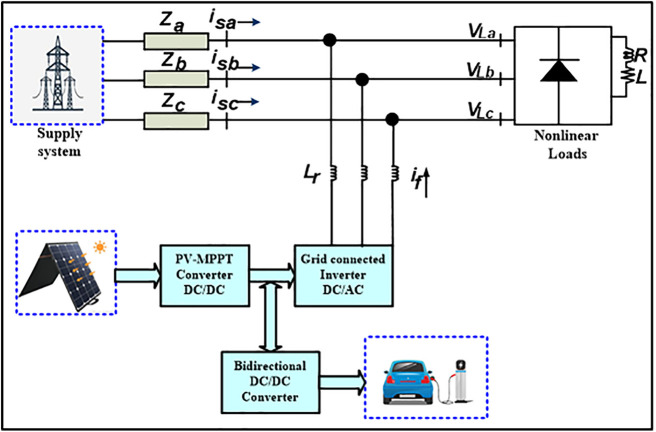
Proposed work with EV charging system.

The design of the proposed system is depicted in Fig 1. The integration of the solar PV and EV systems within the PDS influences the performance of the PQ in the PDS. As a result, this paper employs a SHAPF to mitigate harmonics and enhance the quality of the source current in the PDS. The proposed configuration includes a three-phase PDS, a PV system linked through a DC/DC boost converter, an EV system functioning via a bi-directional converter, and a SHAPF for harmonic compensation and reactive power management. Directly connecting the EV and PV systems on DC is preferable to an AC connection, as it reduces the number of energy conversion stages and improves efficiency. To operate the solar PV array, the MVSSINC MPPT strategy is utilized to maximize power extraction from the solar system.

## Proposed methodology

In this section two different control approaches are introduced for operation of the proposed work. The first one is employed for reference current generation in VSI, and the second one is used to control the maximum power in the PV system. The details are discussed in the subsequent sub-sections.

### VSI control method

Conventionally, the LMS and RLS estimation techniques are used to measure the reference currents. LMS provides a slower convergence rate as comparison to RLS which offers good convergence rate and the performance of the filter is quite superior to that of LMS. But in RLS, the iteration rate is much longer than LMS and requires large storage capacity. Therefore, to overcome these issues, the MLPNN controller is proposed in this paper for reference current generation to provide better active and reactive power management for improving the PQ. The MLPNN employed in this study is trained using datasets generated from both simulation and experimental conditions of the PV–EV integrated system under varying load and irradiation scenarios. A comparative analysis is presented which shows that the proposed MLPNN shows minimum THD and better compensation as comparison to the LMS and RLS under different operating conditions.

Because of its ease of use, learning capabilities, and generalization skills, the MLPNN algorithm has garnered attention recently and is used in various engineering fields.

The key characteristics of this approach include:

- Each neuron pattern within the network utilizes a non-linear differentiable activation function.
- The network comprises multiple hidden layers situated between the input and output nodes.
- The synaptic weights indicate a significant level of connectivity throughout the network.

The dataset consists of input–output pairs representing system variables such as source current, load current, DC-link voltage, and reference compensating currents. A total of approximately 10,000 samples were generated under different operating conditions to ensure robustness. The dataset is divided into 70% for training, 15% for validation and 15% for testing. This split ensures proper generalization and avoids overfitting of the MLPNN model. The learning rate is 0.01 and training time is 10–20 sec. The network is trained using the backpropagation algorithm with gradient descent optimization.

To derive the essential supply current, the VSI needs to either inject or absorb the current from the compensating filter. This compensating current should match the harmonic components and be in phase opposition to them. The model suggested is specifically optimized for high voltage applications within the medium-to-high power range. The subsequent mathematical equations are provided:


$$\begin{array}{c} \begin{array}{c} i_{s(a+b+c)}=i_{sn}\\ i_{L(a+b+c)}=i_{Ln}\\ i_{comp(a+b+c)}=i_{compn} \end{array} \end{array}$$ (1)

Where, the source, load and three-phase compensated currents are reveals as $i_{s(a+b+c)}$, $i_{L(a+b+c)}$ and $i_{comp(a+b+c)}$ respectively. In VSI, $i_{comp(a+b+c)}$ can be shown as the addition of the currents of the two coupling inductors for each phase, as shown below:


$$\begin{array}{c} \begin{array}{c} i_{compa}=i_{La\text{1}}+i_{La\text{2}}={-i}_{La\text{3}}-i_{La\text{4}}\\ i_{compb}=i_{Lb\text{1}}+i_{Lb\text{2}}={-i}_{Lb\text{3}}-i_{Lb\text{4}}\\ i_{compc}=i_{Lc\text{1}}+i_{Lc\text{2}}={-i}_{Lc\text{3}}-i_{Lc\text{4}} \end{array} \end{array}$$ (2)

The equation for dc-link capacitance ${(C}_{dc})$ of VSI is:


$$\begin{array}{c} \begin{array}{ccc} \text{0}.\text{5}\times C_{dc}\times \left[\left(\overset{\text{2}}{\underset{dc}{V}}-\overset{\text{2}}{\underset{dcmin}{V}}\right)\right] & = & V_{s}(t)\times I\times \Delta t \end{array} \end{array}$$ (3)

**Fig 2:**
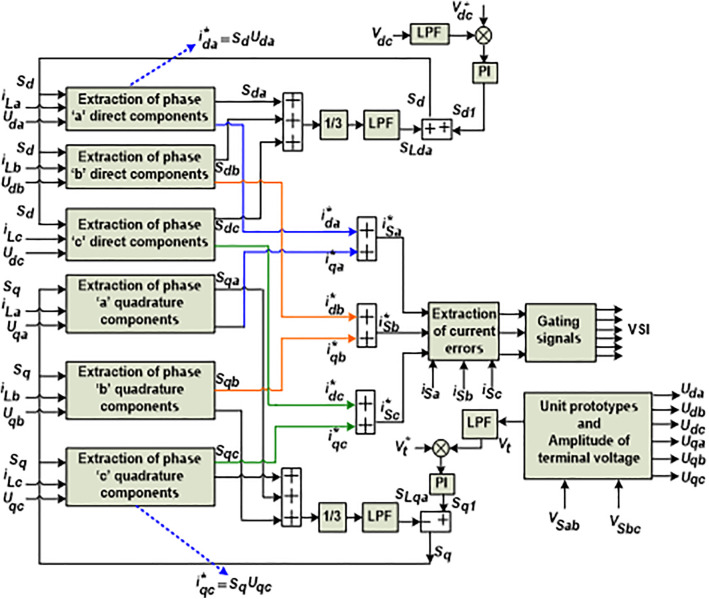
Proposed control strategy.

**Fig 3:**
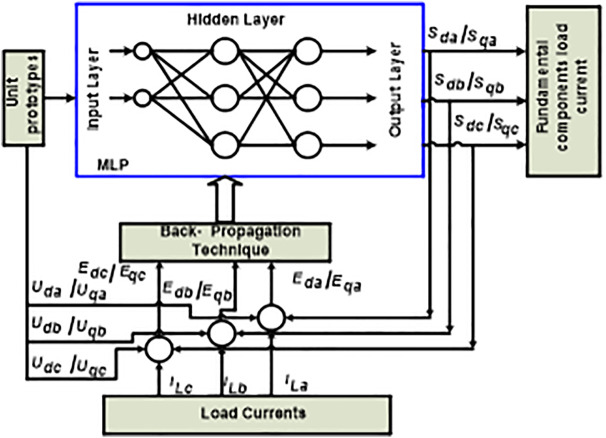
Configuration of MLPNN.

The variables $C_{dc}$, $V_{dc}$, $V_{dcmin}$, I, and Δt that represent the dc-bus capacitance, the dc-side voltage, the lowest dc-bus voltage level, the phase current, the phase voltage, and the time interval needed to increase the dc-bus voltage are identified in this context. The following subsections provide specifics on the MLPNN-based control strategy for the VSI, which is shown in Figs 2 and 3.

### Calculation of supply voltage unit prototypes and maximum amplitude of phase voltages at PCC

The PCC phase voltages at PCC are computed as


$$\begin{array}{c} \begin{array}{ccc} V_{sa} & = & (\text{2}/\text{3})\left(V_{Sab}+V_{Sbc}\right)\\ V_{sb} & = & (\text{2}/\text{3})\left({-V}_{Sab}+V_{Sbc}\right)\\ V_{sc} & = & (\text{2}/\text{3})\left(-V_{Sab}-\text{2}V_{Sbc}\right) \end{array} \end{array}$$ (4)

The load voltage is computed as:


$$\begin{array}{c} V_{t}={\left\{(\text{2}/\text{3})\times \left(\overset{\text{2}}{\underset{Sa}{V}}+\overset{\text{2}}{\underset{Sb}{V}}+\overset{\text{2}}{\underset{Sc}{V}}\right)\right\}}^{\text{1}/\text{2}} \end{array}$$ (5)

Simultaneously, the direct and quadrature unit prototypes (*d, q*) are calculated using (4) and (5),


$$\begin{array}{c} \begin{array}{ccc} U_{da} & = & \frac{V_{Sa}}{V_{t}}\\ U_{db} & = & \frac{V_{Sb}}{V_{t}}\\ U_{dc} & = & \frac{V_{Sc}}{V_{t}} \end{array} \end{array}$$ (6)

And


$$\begin{array}{c} \begin{array}{ccc} U_{qa} & = & \sqrt{\frac{\text{1}}{\text{3}}}\left({-U}_{db}+U_{dc}\right)\\ U_{qb} & = & \frac{\sqrt{\text{3}}}{\text{2}}\left(U_{da}\right)+\text{2}\sqrt{\text{3}}\left(U_{db}-U_{dc}\right)\\ U_{qc} & = & \frac{-\sqrt{\text{3}}}{\text{2}}\left(U_{da}\right)+\text{2}\sqrt{\text{3}}\left(U_{db}-U_{dc}\right) \end{array} \end{array}$$ (7)

### Evaluation of loss components

The synaptic weight of reactive loss component ${(S}_{q\text{1}})$ can be computed from the terminal error voltage, $V_{tE}(k)$.


$$\begin{array}{c} V_{tE}(k)=\overset{*}{\underset{t}{V}}(k)-V_{t}(k) \end{array}$$ (8)

$$\begin{array}{c} S_{q\text{1}}(k+\text{1})=S_{q\text{1}}(k)+K_{p}\left(V_{TE}(k+\text{1})-V_{TE}(k)\right)+{K_{i}V}_{TE}(k+\text{1}) \end{array}$$ (9)

Also, the synaptic weight of active loss component $\left(S_{d\text{1}}\right)$ can be computed from dc-bus error voltage, $V_{dcE}(k)$.


$$\begin{array}{c} V_{dcE}(k)=\overset{*}{\underset{dc}{V}}(k)-V_{dc}(k) \end{array}$$ (10)

$$\begin{array}{c} S_{d\text{1}}(k+\text{1})=S_{d\text{1}}(k)+K_{p}\left(V_{dcE}(k+\text{1})-V_{dcE}(k)\right)+{K_{i}V}_{dcE}(k+\text{1}) \end{array}$$ (11)

And $K_{p}$ and $K_{i}$ in (9) and (11) are proportional and integral gains of the PI controller respectively.

### Extraction of synaptic weights from load currents

The fundamental in-phase weight ${(S}_{da})$ of phase ‘a’ load current is computed as,


$$\begin{array}{c} S_{da}(k+\text{1})=S_{da}(k)+\zeta_{da}(k) \end{array}$$ (12)

$$\begin{array}{c} {\zeta_{da}(k)=\eta }_{d}U_{da}(k)E_{da}(k)\Phi_{da}(k) \end{array}$$ (13)

$$\begin{array}{c} \Phi_{da}(k)=\frac{\text{1}}{\text{1}+{exp}^{-\left\{b+S_{da}(k).U_{da}(k)\right\}}} \end{array}$$ (14)

$$\begin{array}{c} \Phi_{da}(k)=\frac{{exp}^{-\left\{b+S_{da}(k).U_{da}(k)\right\}}}{{\left[\text{1}+{exp}^{-\left\{b+S_{da}(k).U_{da}(k)\right\}}\right]}^{\text{2}}} \end{array}$$ (15)

$$\begin{array}{c} E_{da}(k)=i_{La}(k){-S}_{da}(k).U_{da}(k) \end{array}$$ (16)

Where, $E_{da}$ and $\Phi_{da}$ are the error and activation functions for in-phase components, respectively. The back-propagation algorithm’s learning rate parameter is represented as, $\eta_{d}$ and ‘*b*’ is the activation function’s bias. Similar values are discovered for phases *b* and *c*.

Simultaneously, the synaptic basic quadrature components weight term ${(S}_{qa})$ of phase ‘*a*’ load current is determined as follows:


$$\begin{array}{c} S_{qa}(k+\text{1})=S_{qa}(k)+\zeta_{qa}(k) \end{array}$$ (17)

$$\begin{array}{c} {\zeta_{qa}(k)=\eta }_{q}U_{qa}(k)E_{qa}(k)\Phi_{qa}(k) \end{array}$$ (18)

Where, the error and activation functions for the quadrature components are denoted by, $E_{qa}$ and $\Phi_{qa}$ respectively. The bias imposed in the activation function is “*b*,” and “$\eta_{q}$” is the back-propagation algorithm’s learning rate parameter. Comparable values are discovered for phases B and C.

### Estimation of reference currents

The supply currents in terms of direct and quadrature reference are computed as


$$\begin{array}{c} \begin{array}{c} \overset{*}{\underset{da}{i}}=S_{d}U_{da};\overset{*}{\underset{db}{i}}=S_{d}U_{db};\overset{*}{\underset{dc}{i}}=S_{d}U_{dc}\\ \overset{*}{\underset{qa}{i}}=S_{q}U_{qa};\overset{*}{\underset{qb}{i}}=S_{q}U_{qb};\overset{*}{\underset{qc}{i}}=S_{q}U_{qc} \end{array} \end{array}$$ (19)

Where, $S_{d}=S_{Lda}+S_{dl}$ and $S_{q}=-S_{Lqa}+S_{ql}$ in which $\left\{S_{Lda}=\frac{\left(S_{da}+S_{db}+S_{dc}\right)}{\text{3}}\right\}$ and $\left\{S_{Lqa}=\frac{\left(S_{qa}+S_{qb}+S_{qc}\right)}{\text{3}}\right\}$.

Finally, the reference current is calculated from both direct and quadrature components.


$$\begin{array}{c} \overset{*}{\underset{Sa}{i}}=\overset{*}{\underset{da}{i}}+\overset{*}{\underset{qa}{i}};\overset{*}{\underset{Sb}{i}}=\overset{*}{\underset{db}{i}}+\overset{*}{\underset{qb}{i}};\overset{*}{\underset{Sc}{i}}=\overset{*}{\underset{dc}{i}}+\overset{*}{\underset{qc}{i}} \end{array}$$ (20)

Reference currents are then related with the grid currents, and the output is used to generate the gating signals.

### PV power control MPPT method

#### Framework for PV systems utilizing the GWO-ANFIS algorithm.

MPPT control strategies are utilized to maximize the energy transfer from the photovoltaic system to a specified load via a boost converter. The implemented strategy makes it easier for the boost converters to respond to changing weather conditions on time. in the study, GWO-ANFIS MPPT technique is presented, and its explanation follows in the next part.

#### Controller based on ANFIS.

**Fig 4:**
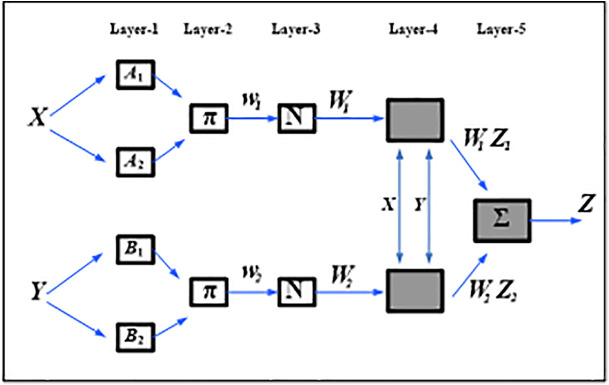
Five layers layout of ANFIS model.

Since the system is operated by a non-linear source (PV array), employing a machine learning technique such as ANFIS is very advantageous. Datasets with input and output variables are used to train it. Two types of these variables are considered: linear and non-linear. Two essential elements of the ANFIS architecture in a fuzzy inference scheme are the antecedent and consequent associated with a rule-based framework. Fig 4 depicts the five-layers adaptive neuro-fuzzy system.

The generalised Gaussian membership function (μ) defines each node’s output in the first layer once it receives the two inputs *x* and *y*. This process is illustrated through Eqs (21)–(23). Here, ${\mu A}_{i}$ and ${\mu B}_{i}$ represents the degrees of membership, the premises parameter set and is represented by


$$\begin{array}{c} {OP}_{\text{1}iL}=\mu_{A_{i}}(x),\;i=\text{1},\text{2}, \end{array}$$ (21)

$$\begin{array}{c} {OP}_{\text{1}iL}=\mu_{B_{i-\text{2}}}(y),\;i=\text{3},\text{4}, \end{array}$$ (22)

$$\begin{array}{c} \mu (x)=e^{{-\left(\frac{x-p_{i}}{a_{i}}\right)}^{\text{2}}} \end{array}$$ (23)

In the subsequent phase, to find the output of every node in the second layer, Eq (24) is used


$$\begin{array}{c} {OP}_{\text{2}iL}=\mu_{A_{i}}(x)\times \mu_{B_{i-\text{2}}}(y) \end{array}$$ (24)

The result produced by the node in the third layer is characterized by Eq (25), and stated as the normalized firing strength, and ${(W}_{I})$ is the firing strength that the third layer produces.


$$\begin{array}{c} {OP}_{\text{3}iL}=W_{I}=\frac{W_{I}}{\sum_{i-\text{1}}^{\text{2}}\overset{\prime }{\underset{i}{W}}} \end{array}$$ (25)

The fourth-layer adaptive node uses the information it receives ${OP}_{\text{3}iL}$from the third layer to decide its own output. As shown in Eq (26), the node’s consequent parameters are represented as $j_{i}$, $k_{i}$ and $l_{i}$. The single node located at the conclusion of the ANFIS model calculates its output using Eq (27).


$$\begin{array}{c} {OP}_{\text{4}iL}=W_{I}f_{i}=W_{I}\left(j_{i}x+k_{i}y+l_{i}\right) \end{array}$$ (26)

$$\begin{array}{c} {OP}_{\text{5}}=\sum if_{i}W_{i} \end{array}$$ (27)

Now from the above equation the ITAE is calculated because it serves as a mechanism to analyse the efficiency of the system.


$$\begin{array}{c} ITAE=\int_{\text{0}}^{\infty }t\left|e(t)\right| \end{array}$$ (28)

#### GWO-based ANFIS controller.

**Fig 5:**
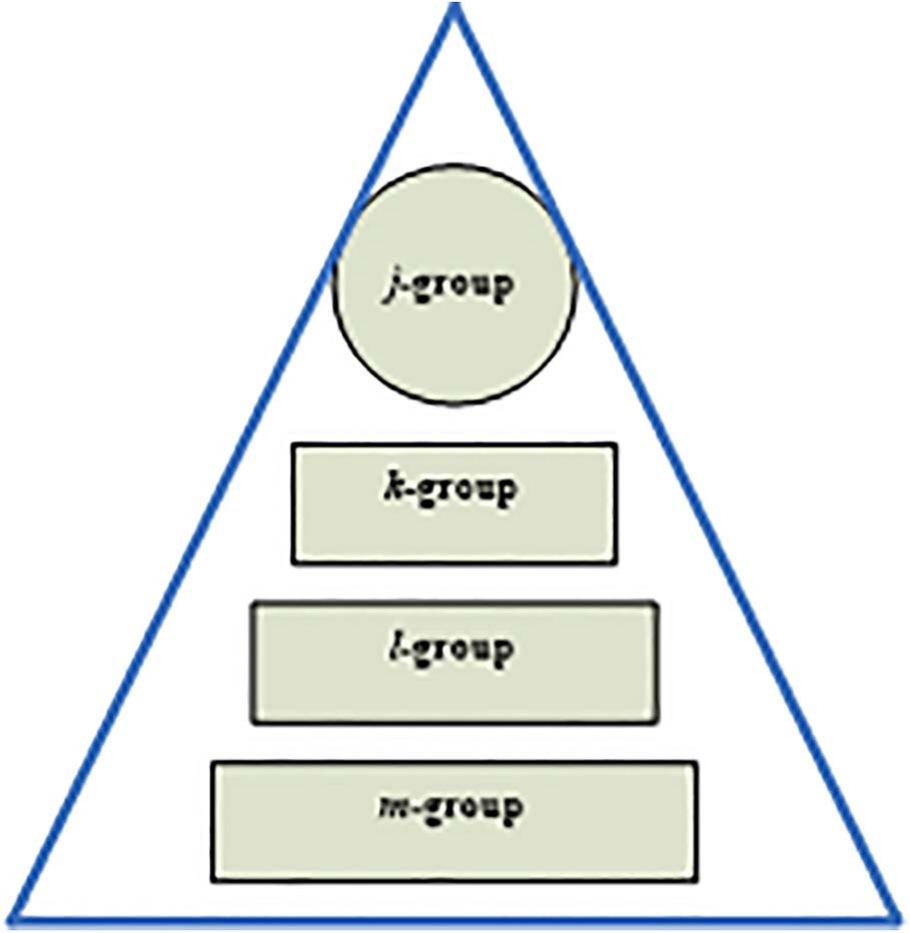
Hierarchy levels of GWO groups.

GWO is a system that mimics how grey wolves interact with each other. Grey wolves usually lived in groups of five to ten. Fig 5 shows their rigidly hierarchical dominance, which is split into groups of grey wolves labelled *j, k, l, and m*.

As the pack leaders, the *j*-group wolves (either male or female) make decisions about where to hunt, when to migrate, when to wake up, and other things. The wolf in the pack that the other wolves fully obey is the *j*-group wolf. Only the *j* wolves in the pack are allowed to mate. When it comes to leading the pack, they are usually successful. Despite their lack of strength, they have gained more importance because of their disciplined and organised behaviour.

**Fig 6:**
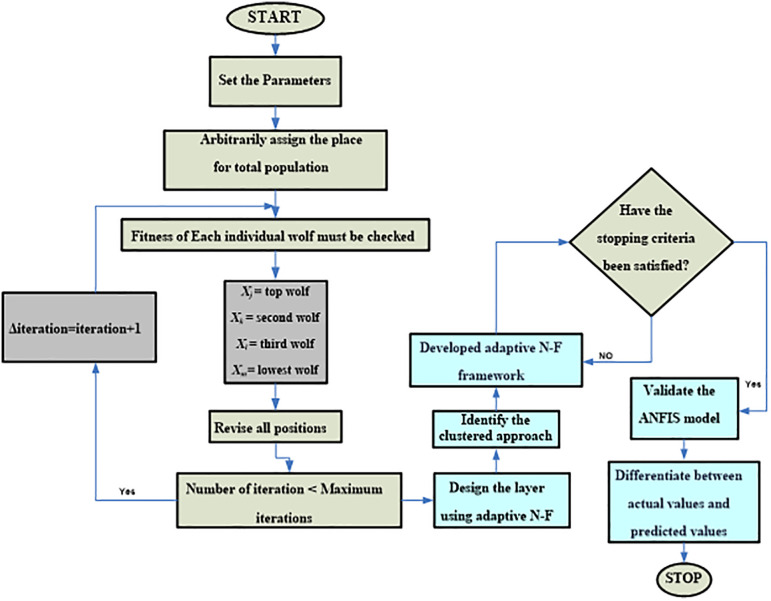
Flowchart of the proposed approach.

On the second level of the hierarchy, the *k*-group can assist the *j*-group wolves with decision-making and other pack duties. The *k*-group wolves become the pack leaders when the *j-*group dies or gets old. Now, the other lower-level wolves are under the *k*-group, but they still owe the *j*-group. The next level up in the hierarchy is the *l-*group, and the last group is the *m*-group. Even though it gives in to *j* and *k* group, the *l* group oversees *m*. The *m*-groups are the pack’s lowest-ranking wolves, so they are always under the control of other leading wolves. The *m-*group wolves are the last to get food and are used as targets by the pack. Regarding the study, the training set includes the following input parameters: PV current (*I*_*PV*_), PV voltage (*V*_*PV*_), ambient temperature (*T*), and solar radiation (*G*) and duty cycle is the output parameter. The FIS, which is produced during training and makes use of the input parameters, rapidly generates the estimated duty. Fig 6 shows the flowchart of the approached control method.

## Result analysis

### MATLAB/Simulation analysis

The proposed power system discussed in Fig 1, is executed using the MATLAB/ Simulation tool. In the proposed system, the PQ is analysed with the use of solar PV array and EV. The main problem arises in the supply current when any kind of non-linear load, PV system or EV system is connected. The main work done in this paper is to analyse the PQ only, rather than analysing the efficiency of PV and EV charging. Due to the integration of non-linear load, PV and EV, the supply current gets distorted and harmonics content becomes high. Therefore, in this work two different controllers are employed for both the grid connected inverter and the MPPT technique for controlling the maximum power.

The MLPNN is employed to produce the reference currents, which are then related with the actual currents to produce the gating pulses. The actual DC voltage is compared to the desired DC voltage, and the resulting error is sent to the ANFIS controller in place of the PI controller to compute the error. The outcomes of the simulation are examined for various waveforms, including source voltage, source currents, load currents, the inverter’s output, and DC link voltages. The harmonics necessary for the non-linear loads are introduced in shunt using a VSI, which is reflected as the compensation current.

### Balanced non-linear loading condition

**Fig 7:**
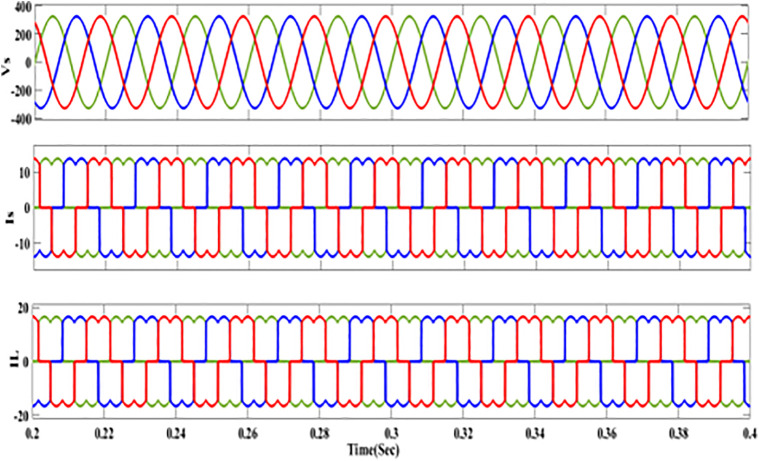
Simulated results of the proposed system before filtering source voltages and currents, load and filter currents.

**Fig 8:**
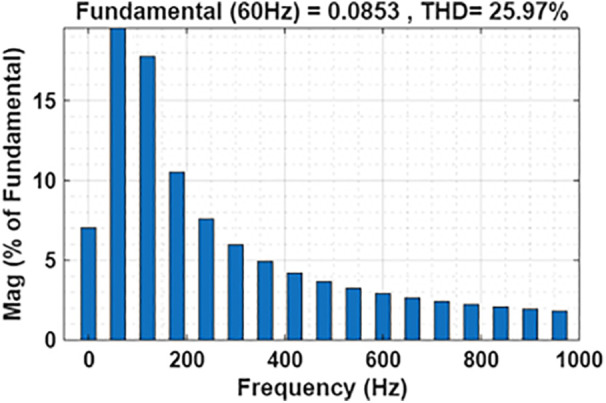
THD value of source current.

The distorted source or load current and the harmonic spectra before filtering are displayed in Fig 7. From Fig 7, noticeably it is marked that the source current THD is 25.97% and is not in compliance with IEEE standards. The voltage and current from the source, as well as the load current for all three phases prior to filtering, are illustrated in Fig 8.

**Fig 9:**
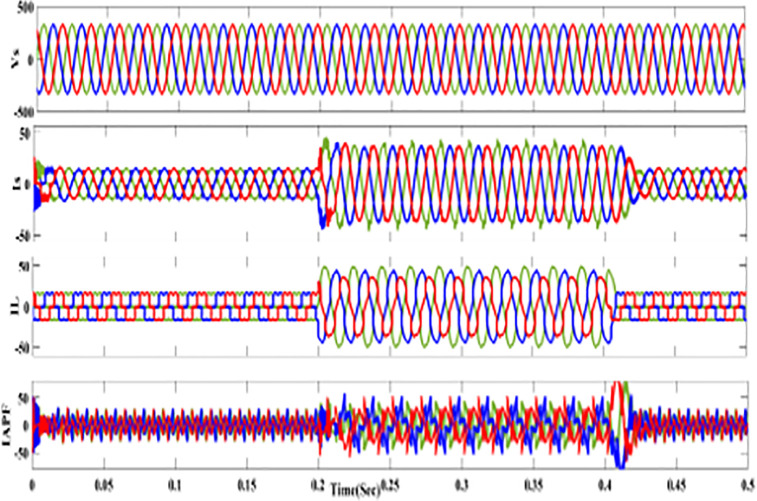
Results obtained during simulation when only SPF connected source voltages and currents, load and filter currents.

**Fig 10:**
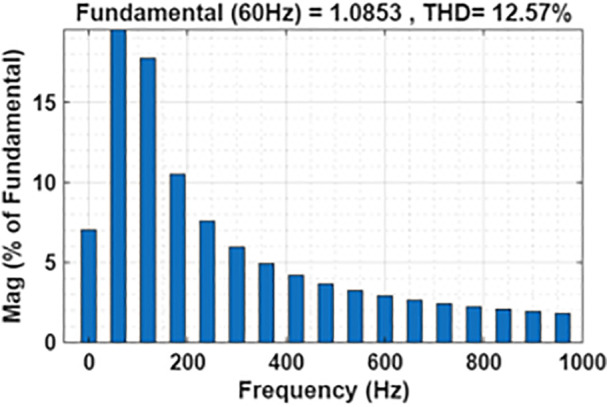
THD value of source current when only SPF connected.

To reduce source current THD and to improve the performance, first SPF is interfaced to the system, and the results are displayed in Fig 9. A reduction of THD from 25.97% to 12.57% is undoubtedly visible from Fig 9. A load variation to evaluate the filter performance is introduced at 0.3 and 0.4 seconds, and different waveforms of the SPF system are presented in Fig 10.

**Fig 11:**
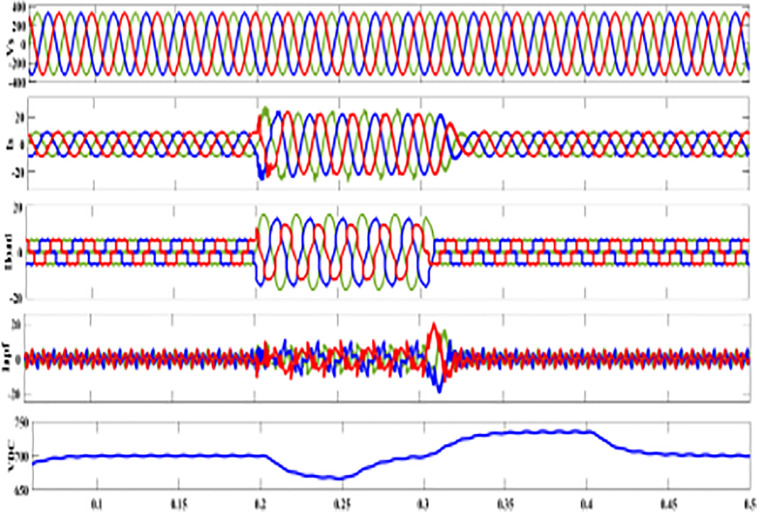
Results obtained during simulation using SHAPF with RLS controller source voltages and currents, load and filter currents, voltage at dc link.

**Fig 12:**
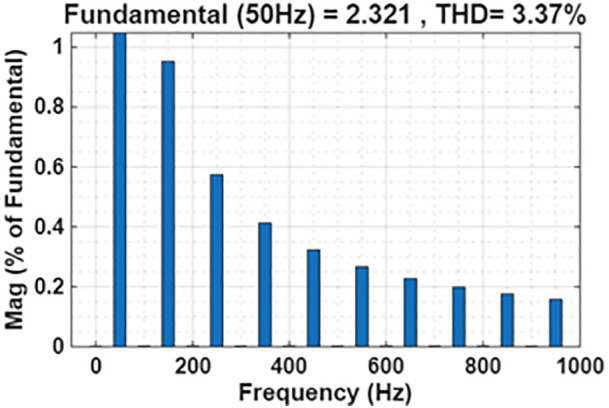
THD value of source current using SHAPF with RLS controller.

Once more, the decrease in THD and the effectiveness of the filter are not adequate. To additionally improve the presentation of the framework, an active filter is embedded to form a hybrid filter. Initially, the source current and other parameters are analysed using RLS algorithm. The simulation results are illustrated in Fig 11. The THD value is shown in Fig 12.

**Fig 13:**
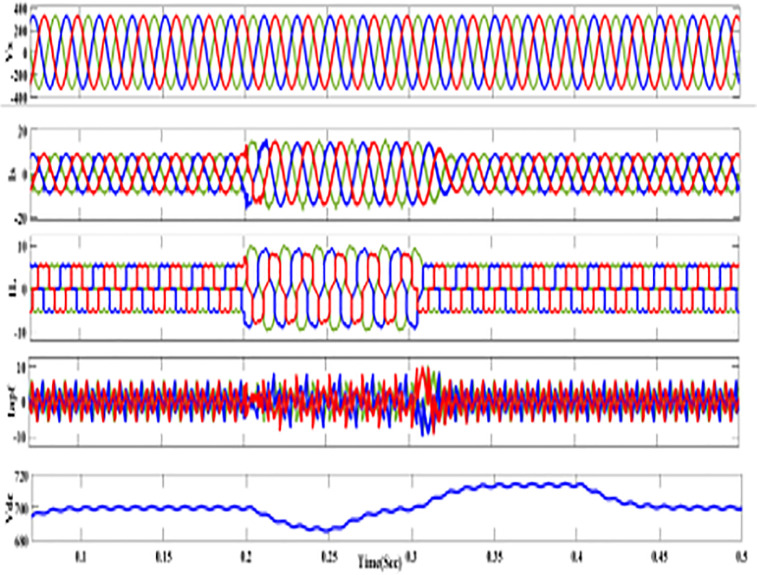
Results obtained during simulation using SHAPF with MLPNN controller source voltages and currents, load and filter currents, voltage at dc link.

**Fig 14:**
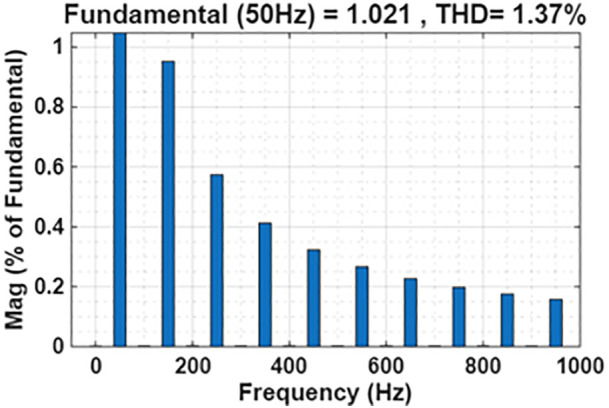
THD value of source current using SHAPF with MLPNN.

Furthermore, the system is observed using the proposed MLPNN method. The simulation waveforms outcomes and THD are illustrated in Figs 13 and 14 respectively. It is evident from the figure that the improvement of harmonics distortions using MLPNN is found relatable better compared to the conventional RLS algorithm.

### Unbalanced nonlinear loading condition

**Fig 15:**
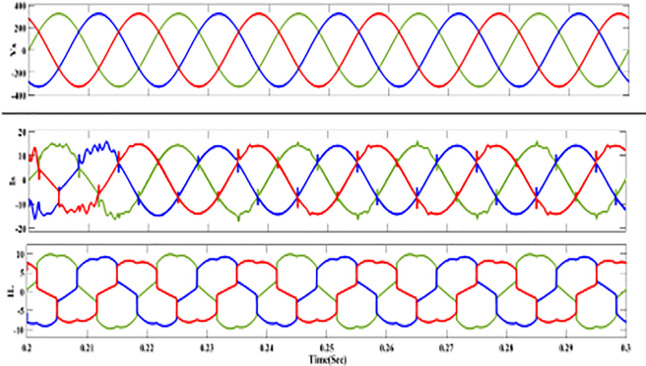
Simulated results of the proposed system before filtering with unbalanced load: Source voltage, source and load current.

In the preceding section, the system’s performance was evaluated under balanced loading conditions, while this section focuses on unbalanced loads. Initially, simulation results are presented without the application of any filters. The harmonic spectrum of the three-phase source and load currents (*phase-a, phase-b, phase-c*), as well as the source voltage, are shown in Fig 15.

**Fig 16:**
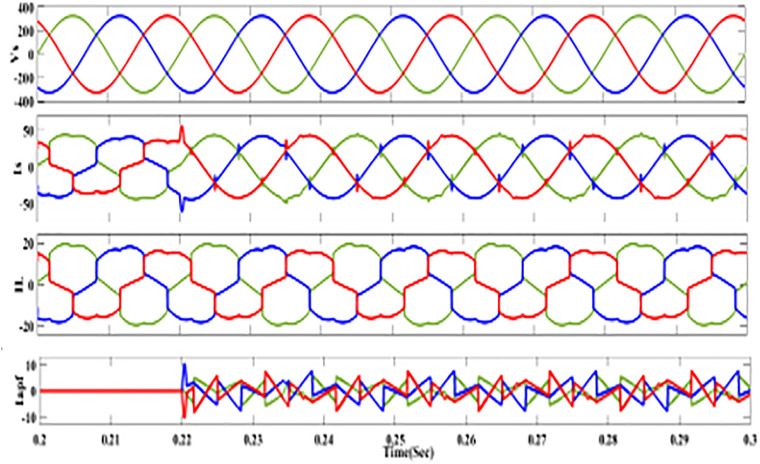
Results obtained during simulation when only SPF is connected to the proposed system with unbalanced load: (d) Source voltage and current, load and filter current.

The figure clearly illustrates that the peak values of the individual load currents vary, with their RMS values for phases a, b, and c, respectively. To improve power quality in the presence of an unbalanced load, an analysis was conducted using the SPF and SHAPF techniques, employing RLS and MLPNN methods. The simulation results showcasing various waveforms comprising the source voltage, load and supply currents, as well as source current with the SPF are examined in Fig 16. Upon connecting the SPF, the %THD of the source currents showed improvement (though not entirely satisfactory), recorded as 8.08%, 8.65%, and 8.66% for the three phases, respectively. However, the RMS values of the source currents varied, measuring 19.5A, 13.98A, and 20.17A, respectively. The harmonic spectrum for the three-phase currents (*phase-a, phase-b, phase-c*) can be observed in Fig 16.

**Fig 17:**
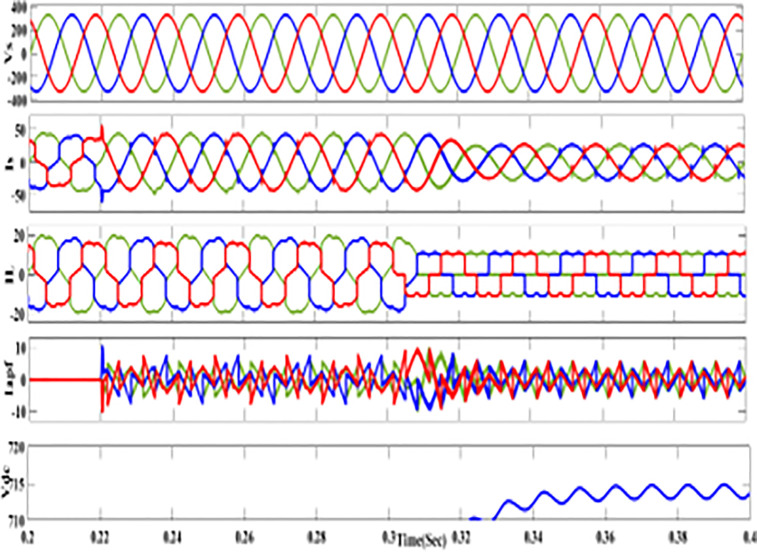
Simulation results of RLS-SHAPF System with unbalanced load: Source voltage, source and load current, filters currents, voltage at dc link.

In the subsequent case, the suggested system utilizes RLS-based SHAPF. To evaluate the resilience of the system, the SPF and SHAPF are activated sequentially at t = 0.4s and t = 0.5s. The results can be observed in Fig 17. From Fig 17, it is evident that after t = 0.5s, when SHAPF is activated, the source current becomes sinusoidal and balanced. The THD percentages are recorded at 3.44%, 4.20%, and 3.32% respectively. Additionally, the RMS values for the three phases are measured at 17.2A, 17.08A, and 17.41A respectively.

**Fig 18:**
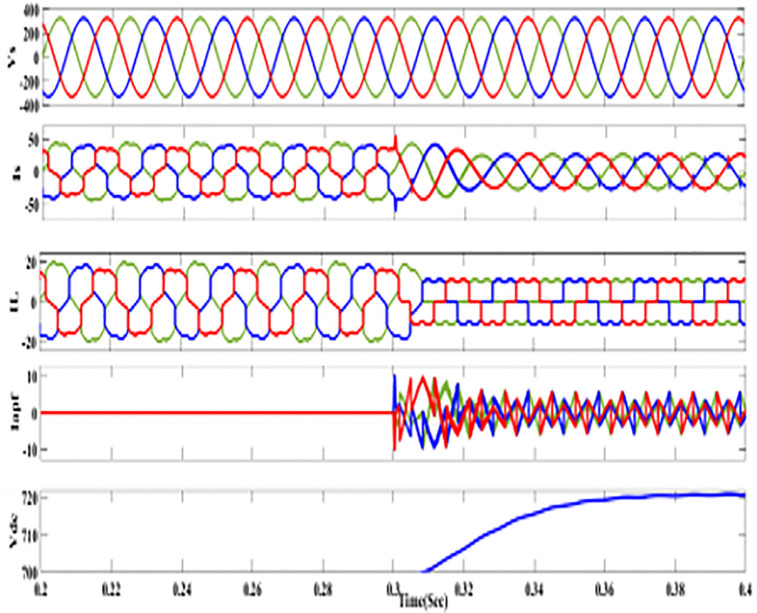
Simulation results by MLPNN based SHAPF with unbalanced load: Source voltage, source and load current, filters currents, voltage at dc link.

Fig 17 illustrates the harmonic analysis of the source current, along with the waveforms of the source voltage, source and load currents, filter currents, and the voltage at the DC link for the RLS-based SHAPF system. A comparable analysis has been performed for the MLPNN-SHAPF system, and the waveforms are shown in Fig 18.

In comparison to the traditional RLS, the MLPNN-based SHAPF demonstrates enhanced performance regarding THD, settling time, voltage ripple, and other parameters. The values for all parameters recorded during the simulation across various loading conditions are presented in Table 2.

**Table 2:** System parameters.

Parameters	Values
Grid Line Voltage (RMS)	415 V
Grid Frequency	50 Hz
Switching Frequency	10000 kHz
Load Resistance	10-20 Ω
Load Inductance	5-10 mH
PV array	1000 w
Bi-directional buck boost inductor	2.16mH
DC link voltage	189V
DC link capacitor	1700µF
Filter inductor	1.41mH
Filter capacitor	19.8 µF

### Experimental studies

**Fig 19:**
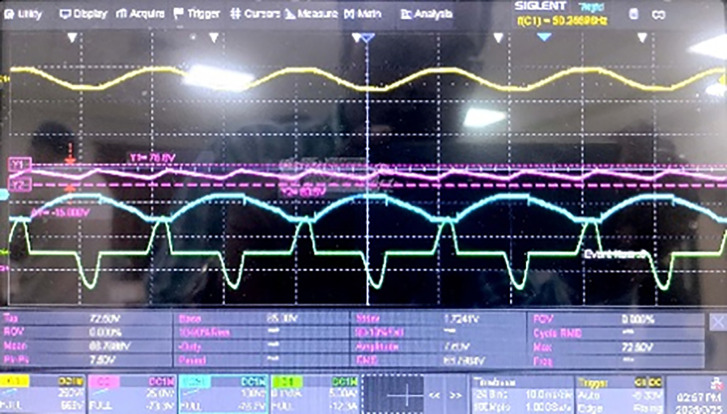
Experimental waveform of source voltage and current before compensation.

**Fig 20:**
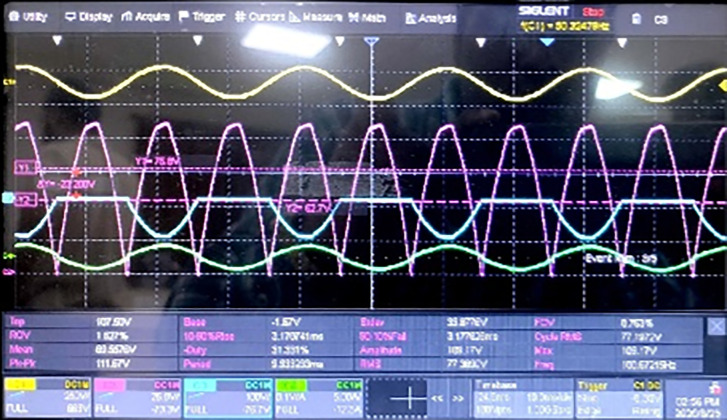
Experimental waveform of source voltage and current after compensation.

To validate the effectiveness of the proposed MLPNN-based active power filtering technique, a real-time experimental setup was developed using a dSPACE 1103 DSP controller. The system operates under a three-phase supply of 415 V, 50 Hz, feeding non-linear loads consisting of a diode bridge rectifier with RL load. The control algorithm MLPNN is implemented in MATLAB/Simulink and interfaced with the dSPACE platform for real-time execution. Voltage and current signals are sensed using Hall-effect sensors (LEM25P and LA55-P) and fed to the ADC channels of the dSPACE controller. The generated switching pulses are applied to the VSI through digital output ports. A multi-channel digital storage oscilloscope (SIGLENT SDS1104X-E-5100) is used for waveform acquisition and analysis. The system performance is evaluated under variable conditions: without compensation, with compensation using SPF, with compensation using SHAPF with RLS and with MLPNN technique. The suggested system’s schematic design is depicted in Figs 19 and 20. The DSO is used to record the sources of voltage and current as well as harmonic reduction data. The suggested MLPNN algorithm manages the PV generating system in real-time by operating on the DSP controller (dSPACE 1103). From the DACs, these detected signals are sent to the dSPACE 1103’s ADCs. The dSPACE 1103’s digital input pins receive the firing pulses for the VSI that are produced by the MLPNN algorithm. The proposed system is analyzed under balanced non-linear loading conditions.

Fig 19 displays the three-phase source voltage and source current experimental waveforms with and without the VSI. Fig 19 shows the distorted source current. During the connection of the non-linear load, the % THD found to be 25.97%, further after connecting the loads and running with the VSI using MLPNN technique, the THD of the source current is reduced to 1.25%. The source current waveform becomes nearly sinusoidal after compensation, indicating effective harmonic mitigation. The settling time, overshoot and dc link ripple voltage are observed in Fig 20 and comparative analysis is provided in Table 3.

**Table 3:** Comparative analysis without and with compensation.

Method	Settling Time (s)	Overshoot (%)	DC-Link Ripple (±V)	Dynamic Response
Without compensation	0.12	14.5	18	Slow
With compensation using MLPNN	0.03	2.1	3	Fast

From the table, it is observed that the proposed MLPNN controller significantly enhances DC-link voltage stability and dynamic response, achieving faster settling time, minimal overshoot, and reduced voltage ripples. These results confirm that the proposed MLPNN-based control strategy significantly enhances PQ and satisfies harmonic standards.

## Limitations and future scope

Even though the effectiveness of the proposed MLPNN-based control strategy in improving PQ, certain limitations must be acknowledged. Firstly, the implementation of the MLPNN controller introduces additional computational complexity compared to conventional RLS algorithms. The inclusion of several neurons, hidden layers, and parameter updates makes the computation more demanding, which may make it difficult to implement the algorithm in real time when employing low-cost controllers. Second, the effectiveness of the suggested method relies heavily on the architecture of the neural network used and the manner in which training is done. Inconsistency in the adjustment of parameters and inadequate training samples could lead to slower convergence rates and imprecise compensation for harmonics. Third, the system is vulnerable to changes in operational conditions like varying load conditions, varying sunlight intensity, and grid instability. Additionally, only a localized grid-connected PV–EV system is included in this analysis. More research is needed to determine whether the suggested approach can be scaled to large-scale distribution networks or smart grids with numerous dispersed energy resources, especially in terms of coordination, communication, and computational load.

Future research will focus on creating neural network models that are computationally efficient, investigating hybrid intelligent control schemes, and extending the proposed methodology to large-scale smart grid settings.

## Conclusion

The increased adoption of PV and EV technology in modern electrical grids represents an essential step in achieving sustainability goals in both power production and transportation. Nevertheless, this development poses a substantial threat to the maintenance of power quality (PQ) due to the inherent variability in PV electricity production and the highly unpredictable charging characteristics of EVs. It should be noted that the level of deterioration of power quality, especially concerning current distortion, will largely depend on the strength of the infrastructure used.

The present paper extensively analyzes the combined impact of using PV systems along with EV on PQ under both balanced and unbalanced load conditions. The findings reveal that a high degree of penetration adversely affects PQ by creating high harmonics in addition to grid parameters. However, this paper also proves that such an adverse effect can be overcome by adopting effective operating and control techniques, which will ensure a reliable power flow to the load along with green charging stations for EVs.

To address these problems, two control approaches, namely Recursive Least Squares (RLS) and Multi-Layer Perceptron Neural Network (MLPNN), were employed for regulating the grid-connected inverter and maximizing power production in photovoltaic cells. The efficiency of the system was tested under two conditions, with and without compensation filter, with emphasis on crucial factors such as DC-link voltage, source voltage, source current, and load current. According to the results obtained, it can be inferred that the use of an intelligent approach is very beneficial in ensuring efficient power quality.

From the comparative analysis, the control system utilizing the MLPNN approach performs better compared to the RLS-based technique in terms of reducing the THD under balanced and unbalanced scenarios. The results illustrate the potential of utilizing adaptive and data-driven techniques to counter the issues arising from the nonlinearities and uncertainties in PV-EV systems. In conclusion, it can be stated that the utilization of the MLPNN technique alongside effective filtering techniques could significantly contribute to improving PQ levels, thus ensuring efficient integration of renewable sources and EVs in future smart grids.
